# O Fenômeno de "*No Reflow*" Muito Além da Sala de Hemodinâmica

**DOI:** 10.36660/abc.20250782

**Published:** 2026-03-13

**Authors:** Antonio Hélio Pozetti, Henrique Barbosa Ribeiro

**Affiliations:** 1 Hospital Austa São José Rio Preto SP Brasil Hospital Austa São Josédo Rio Preto, SP – Brasil; 2 Universidade de São Paulo Instituto do Coração Faculdade de Medicina São Paulo SP Brasil Instituto do Coração do Hospital das Clínicas da Faculdade de Medicina da Universidade de São Paulo, São Paulo, SP – Brasil; 3 Hospital Sírio Libanês São Paulo SP Brasil Hospital Sírio Libanês, São Paulo, SP – Brasil

**Keywords:** Fenômeno de No-Reflow, Índice Aterogênico Plasmático, Enxerto de Veia Safena, Intervenção Coronária Percutânea

A doença cardiovascular (DCV) decorrente da aterosclerose, especialmente na forma de síndrome coronariana aguda (SCA), permanece sendo uma das principais causas de morbimortalidade global.¹ A intervenção coronária percutânea (ICP) constitui a estratégia de revascularização de escolha no cenário da SCA.² Apesar dos avanços técnicos e tecnológicos das últimas décadas, especialmente nos casos agudos, a ICP ainda está associada a complicações relevantes, entre as quais se destaca o fenômeno de "*no-reflow*" (FNR).² Sua incidência pode ultrapassar 20% nas angioplastias realizadas durante o infarto agudo do miocárdio com supradesnivelamento do segmento ST.³

O FNR se caracteriza pela incapacidade de restabelecer perfusão miocárdica microvascular adequada, mesmo após a recanalização bem-sucedida do vaso epicárdico.^4^ Trata-se de um marcador robusto de prognóstico adverso, associado a maior extensão de necrose, pior função ventricular esquerda e maior mortalidade no curto e no longo prazo.^4,5^ A fisiopatologia é complexa e multifatorial, envolvendo disfunção endotelial e microvascular, inflamação, embolização distal, formação de trombo e vasoespasmo.^5,6^ Diversos fatores clínicos, angiográficos e relacionados ao procedimento aumentam o risco de FNR — incluindo maior tempo de isquemia, elevada carga trombótica, intervenções em enxertos de veia safena (EVS), níveis elevados de troponina, uso de balões de alta pressão, stents mais longos e marcadores inflamatórios como leucocitose, proteína C reativa e fibrinogênio.^7,8^

Nesse contexto, o índice aterogênico plasmático (IAP), calculado pelo logaritmo da razão entre triglicerídeos e colesterol HDL (log[TG/HDL-c]), tem emergido como marcador substituto de dislipidemia aterogênica, de disfunção endotelial e de inflamação vascular.^9^ Valores elevados de IAP correlacionam-se com maior risco cardiovascular, extensão da doença arterial coronária e maior propensão a complicações microvasculares.^8,9^

Nesta edição da revista, Erdoğan et al.^10^ avaliaram a relação entre IAP e FNR em 287 pacientes submetidos à ICP em lesões de EVS no contexto de infarto sem supradesnivelamento do segmento ST. A taxa de FNR observada (29,3%) foi superior aos ∼20% usualmente descritos na literatura para ICP em EVS.¹¹ Os pacientes que evoluíram com FNR eram mais idosos e apresentavam maior prevalência de comorbidades, incluindo diabetes, fibrilação atrial, doença renal crônica, insuficiência cardíaca e história prévia de acidente vascular cerebral/ataque isquêmico transitório.

Laboratorialmente, esses pacientes apresentaram níveis mais baixos de HDL-c e mais altos de triglicerídeos, o que resultou em aumento significativo do IAP. No grupo com FNR, se observou maior frequência de degeneração do EVS. Na análise multivariada, os preditores independentes de FNR incluíram: EVS degenerado, presença de trombo, fração de ejeção reduzida, maior diâmetro de stent e IAP elevado.^10^ Esses achados reforçam fatores de risco já conhecidos para o FNR^7,8^ e corroboram o papel do IAP como marcador inflamatório e aterogênico associado ao FNR em intervenções em EVS.

O principal destaque do estudo é a identificação de um marcador pré-procedimento, simples e de baixo custo, capaz de auxiliar na estratificação de risco do FNR. O IAP elevado reflete o predomínio de lipoproteínas ricas em triglicerídeos, formação de partículas pequenas e densas de LDL e consequente maior propensão ao estresse oxidativo, à inflamação e à disfunção endotelial.^9^ Como a dosagem direta dessas partículas não é amplamente disponível, o IAP apresenta-se como alternativa factível e clinicamente relevante.

Curiosamente, os valores de LDL-c estavam acima das metas recomendadas pelas diretrizes atuais,^12^ mas sem diferença entre os grupos. Tal achado reforça que o tipo e o comportamento metabólico das partículas de LDL podem ser mais determinantes do que seus valores absolutos, o que justifica a utilidade de marcadores como o IAP. No presente estudo, as medianas de IAP foram relativamente elevadas em ambos os grupos (1,4 e 1,9, respectivamente, para os grupos sem FNR e com FNR), sugerindo um perfil lipídico altamente aterogênico nessa população.

O estudo confirma ainda o papel clássico da carga trombótica e da degeneração do EVS como fortes preditores de FNR.^7,10^ Apesar de as diretrizes recomendarem o uso de dispositivos de proteção embólica (DPE) em intervenções em EVS, a taxa observada foi extremamente baixa (4,8%). Na literatura, sua utilização não ultrapassa 20%–25%^13^ e tem declinado ao longo dos anos, em razão de limitações técnicas, custos e evidências inconsistentes quanto a um impacto clínico robusto.^13,14^ Estratégias alternativas mais simples, implante direto de stent, stent subdimensionado, inibidores potentes de P2Y12 e vasodilatação intracoronária prévia, demonstraram reduzir o risco de FNR e podem explicar a menor dependência dos DPE.^13,14^

Embora a ICP em EVS siga sendo desafiadora, dados recentes do único estudo randomizado comparando ICP em vasos nativos versus EVS, conduzido por Winter et al.,^15^ demonstraram menor taxa de infarto periprocedimento e menor necessidade de revascularização do vaso-alvo em um ano no grupo tratado no enxerto venoso — embora mais de 80% das lesões nativas eram de oclusões crônicas. Notavelmente, as taxas de FNR foram inferiores ao esperado no grupo EVS, reforçando que, em anatomias complexas, a ICP do enxerto pode ser uma estratégia adequada e segura.

## Considerações finais

A inflamação desempenha um papel central na aterogênese e no risco de complicações microvasculares. O estudo de Erdoğan et al.^10^ fornece evidência adicional de que o IAP, um marcador simples, reprodutível e amplamente disponível, pode auxiliar na identificação de pacientes com maior risco de FNR em intervenções em EVS. Apesar das limitações inerentes ao desenho retrospectivo, observacional e unicêntrico, os achados são coerentes e relevantes para a prática.

A tomada de decisão na ICP de EVS deve ir além das características angiográficas, incorporando um julgamento integrado que inclua aspectos clínicos, metabólicos (incluindo marcadores de aterogenicidade e inflamação), angiográficos e do procedimento. Essa abordagem multidimensional tem potencial para otimizar os resultados da revascularização e reduzir eventos adversos no tratamento percutâneo da doença coronária.

## References

[1] Martin SS, Aday AW, Almarzooq ZI, Anderson CAM, Arora P, Avery CL (2024). 2024 Heart Disease and Stroke Statistics: A Report of US and Global Data from the American Heart Association. Circulation.

[2] Byrne RA, Rossello X, Coughlan JJ, Barbato E, Berry C, Chieffo A (2023). 2023 ESC Guidelines for the Management of Acute Coronary Syndromes. Eur Heart J.

[3] Jaffe R, Charron T, Puley G, Dick A, Strauss BH (2008). Microvascular Obstruction and the No-Reflow Phenomenon after Percutaneous Coronary Intervention. Circulation.

[4] Kloner RA, Ganote CE, Jennings RB (1974). The "No-Reflow" Phenomenon after Temporary Coronary Occlusion in the Dog. J Clin Invest.

[5] Pelliccia F, Niccoli G, Zimarino M, Andò G, Porto I, Calabrò P (2023). Pathophysiology and Treatment of the No-Reflow Phenomenon in ST-Segment Elevation Myocardial Infarction: Focus on Low-Dose Fibrinolysis during Primary Percutaneous Intervention. Rev Cardiovasc Med.

[6] Niccoli G, Scalone G, Lerman A, Crea F (2016). Coronary Microvascular Obstruction in Acute Myocardial Infarction. Eur Heart J.

[7] Rezkalla SH, Stankowski RV, Hanna J, Kloner RA (2017). Management of No-Reflow Phenomenon in the Catheterization Laboratory. JACC Cardiovasc Interv.

[8] Refaat H, Tantawy A, Gamal AS, Radwan H (2021). Novel Predictors and Adverse Long-Term Outcomes of No-Reflow Phenomenon in Patients with Acute ST Elevation Myocardial Infarction Undergoing Primary Percutaneous Coronary Intervention. Indian Heart J.

[9] Assempoor R, Daneshvar MS, Taghvaei A, Abroy AS, Azimi A, Nelson JR (2025). Atherogenic Index of Plasma and Coronary Artery Disease: A Systematic Review and Meta-Analysis of Observational Studies. Cardiovasc Diabetol.

[10] Erdoğan O, Panç C (2025). Valor Preditivo do Índice Aterogênico para Não-Refluxo em Pacientes Submetidos à ICP para Enxerto de Veia Safena na Síndrome Coronariana Aguda. Arq Bras Cardiol.

[11] Muller O, Windecker S, Cuisset T, Fajadet J, Mason M, Zuffi A (2008). Management of Two Major Complications in the Cardiac Catheterisation Laboratory: The No-Reflow Phenomenon and Coronary Perforations. EuroIntervention.

[12] Vrints C, Andreotti F, Koskinas KC, Rossello X, Adamo M, Ainslie J (2024). 2024 ESC Guidelines for the Management of Chronic Coronary Syndromes. Eur Heart J.

[13] Paul TK, Bhatheja S, Panchal HB, Zheng S, Banerjee S, Rao SV (2017). Outcomes of Saphenous Vein Graft Intervention with and without Embolic Protection Device: A Comprehensive Review and Meta-Analysis. Circ Cardiovasc Interv.

[14] Brennan JM, Al-Hejily W, Dai D, Shaw RE, Trilesskaya M, Rao SV (2015). Three-Year Outcomes Associated with Embolic Protection in Saphenous Vein Graft Intervention: Results in 49 325 Senior Patients in the Medicare-Linked National Cardiovascular Data Registry CathPCI Registry. Circ Cardiovasc Interv.

[15] Winter RW, Hoek R, Walsh SJ, Hanratty CG, Sprengers RW, Twisk JWR (2025). PCI of Native Coronary Artery vs Saphenous Vein Graft after Prior Bypass Surgery: A Multicenter, Randomized Trial. J Am Coll Cardiol.

