# Supplementary material for: O Fenômeno de "No Reflow" Muito Além da Sala de Hemodinâmica
Source: Arq Bras Cardiol. 2026 Mar 13;122(11):e20250782. [Article in Portuguese] doi: 10.36660/abc.20250782 (PMC13361802; doi:10.36660/abc.20250782)
Supplement: Translation

# Translation: The "No-Reflow" Phenomenon Far Beyond the Cath Lab

**DOI:** 10.36660/abc.20250782i

**Authors:** Antonio Hélio Pozetti, Henrique Barbosa Ribeiro

**Affiliations:** 1 Rio Preto SP Brazil Hospital Austa São José do Rio Preto, SP – Brazil; 2 São Paulo SP Brazil Instituto do Coração do Hospital das Clínicas da Faculdade de Medicina da Universidade de São Paulo, São Paulo, SP – Brazil; 3 São Paulo SP Brazil Hospital Sírio Libanês, São Paulo, SP – Brazil

**Keywords:** No-Reflow Phenomenon, Atherogenic Index of Plasma, Saphenous Vein Graft, Percutaneous Coronary Intervention

Cardiovascular disease (CVD) secondary to atherosclerosis, particularly in the form of acute coronary syndrome (ACS), remains one of the leading causes of global morbidity and mortality.^1^ Percutaneous coronary intervention (PCI) is the revascularization strategy of choice in the ACS setting.^2^ Despite meaningful technological advances, PCI continues to be associated with clinically significant complications, of which the no-reflow phenomenon (NRP) is particularly relevant.^2^ Its incidence may exceed 20% in interventions performed during ST-segment elevation myocardial infarction.^3^

NRP is defined as the failure to restore adequate microvascular myocardial perfusion despite successful epicardial vessel recanalization.^4^ It is a strong marker of adverse outcomes and is associated with larger infarct size, impaired left ventricular function, and increased mortality.^4,5^ Its mechanisms are multifactorial, involving microvascular dysfunction, inflammation, distal embolization, thrombus formation, and vasospasm.^5,6^ Several clinical, angiographic, and procedural factors increase NRP risk, including prolonged ischemia, high thrombus burden, saphenous vein graft (SVG) interventions, elevated troponin levels, high-pressure balloon inflations, long stents, and inflammatory markers such as leukocytosis, C-reactive protein, and fibrinogen.^7,8^

Within this context, the atherogenic index of plasma (AIP), calculated as log(TG/HDL-c), has emerged as a surrogate marker of atherogenic dyslipidemia, endothelial dysfunction, and vascular inflammation.^9^ Elevated AIP correlates with higher cardiovascular risk, greater coronary disease extent, and a higher probability of microvascular complications.^8,9^

In this issue, Erdoğan et al.^10^ evaluated the relationship between AIP and NRP in 287 patients undergoing PCI for SVG lesions in non-ST-segment elevation myocardial infarction. The observed NRP rate (29.3%) was higher than the ∼20% typically reported.^11^ Patients with NRP were older and had a higher prevalence of comorbidities, including diabetes, atrial fibrillation, chronic kidney disease, heart failure, and prior stroke/ transient ischemic attack.

These patients also had lower HDL-c and higher triglycerides, resulting in significantly elevated AIP. SVG degeneration was more frequent in the NRP group. Independent predictors of NRP included degenerated SVG, thrombus presence, reduced ejection fraction, larger stent diameter, and elevated AIP.^10^ These findings reaffirm known NRP^7,8^ risk factors and support AIP as an inflammatory and atherogenic marker associated with NRP in SVG interventions.

A key contribution of the study is the identification of a simple, low-cost pre-procedural marker to aid in NRP risk stratification. High AIP reflects the predominance of triglyceride-rich lipoproteins and small, dense LDL particles, which favor oxidative stress, inflammation, and endothelial dysfunction.^9^ As direct measurement of these particles is not broadly accessible, AIP is a feasible and clinically relevant surrogate.

Interestingly, LDL-c values were above guideline-recommended targets^12^ but did not differ between groups. This suggests that the quality and metabolic profile of LDL particles may be more relevant than absolute levels, underscoring the usefulness of markers such as AIP. Median AIP values were relatively high in both groups (1.4 and 1.9), indicating a highly atherogenic lipid profile.

The study also reinforces the classical association between thrombus burden and SVG degeneration with NRP.^7-10^ Although embolic protection devices (EPD) are recommended for SVG interventions, usage was low (4.8%). In the literature, use rarely exceeds 20–25%^13^ and has declined due to technical challenges, costs, and limited evidence of substantial clinical benefit.^13,14^ Simpler alternative strategies, direct stenting, mild undersizing, potent P2Y12 inhibitors, and pre-procedural intracoronary vasodilators, may mitigate NRP risk and reduce dependence on EPDs.^13,14^

Despite the challenges of SVG PCI, the randomized study by Winter et al.^15^ demonstrated lower periprocedural myocardial infarction and one-year target-vessel revascularization rates in the SVG-treated group, even though more than 80% of native vessels had chronic total occlusions. Notably, NRP rates were lower than expected, suggesting that in complex anatomy, SVG PCI may constitute a safe and reasonable strategy.

## Final considerations

Inflammation plays a central role in atherogenesis and microvascular complications. The study by Erdoğan et al.^10^ provides further evidence that AIP, a simple, reproducible, and widely available biomarker, may help identify patients at higher risk of NRP in SVG interventions. Despite the limitations of a retrospective, observational, single-center study, the findings are coherent and clinically relevant.

Decision-making in SVG PCI should integrate clinical, metabolic, angiographic, and procedural factors. A multidimensional approach may optimize revascularization outcomes and reduce adverse events in PCI.
